# Hypermethylation of CCND2 May Reflect a Smoking-Induced Precancerous Change in the Lung

**DOI:** 10.1155/2011/950140

**Published:** 2011-03-28

**Authors:** Alexander Salskov, Stephen E. Hawes, Joshua E. Stern, Qinghua Feng, C. Diana Jordan, Linda Wiens, Janet Rasey, Hiep Lu, Nancy B. Kiviat, Hubert Vesselle

**Affiliations:** ^1^Division of Nuclear Medicine, Department of Radiology, Box 357115, School of Medicine, University of Washington, Seattle, WA 98195-7115, USA; ^2^Department of Epidemiology, School of Public Health, University of Washington, Seattle, WA 98195-7230, USA; ^3^Department of Pathology, School of Medicine, University of Washington, Seattle, WA 98195-6100, USA; ^4^Department of Radiation Oncology, School of Medicine, University of Washington, Seattle, WA 98195-9441, USA

## Abstract

It remains unknown whether tobacco smoke induces DNA hypermethylation as an early event in carcinogenesis or as a late event, specific to overt cancer tissue. Using MethyLight assays, we analyzed 316 lung tissue samples from 151 cancer-free subjects (121 ever-smokers and 30 never-smokers) for hypermethylation of 19 genes previously observed to be hypermethylated in nonsmall cell lung cancers. Only APC (39%), CCND2 (21%), CDH1 (7%), and RARB (4%) were hypermethylated in >2% of these cancer-free subjects. CCND2 was hypermethylated more frequently in ever-smokers (26%) than in never-smokers (3%). CCND2 hypermethylation was also associated with increased age and upper lobe sample location. APC was frequently hypermethylated in both ever-smokers (41%) and never-smokers (30%). BVES, CDH13, CDKN2A (p16), CDKN2B, DAPK1, IGFBP3, IGSF4, KCNH5, KCNH8, MGMT, OPCML, PCSK6, RASSF1, RUNX, and TMS1 were rarely hypermethylated (<2%) in all subjects. Hypermethylation of CCND2 may reflect a smoking-induced precancerous change in the lung.

## 1. Introduction

Lung cancer causes more deaths in the United States each year than breast, colon, pancreatic, and prostate cancer combined, approximately 157,300 deaths estimated in 2010 [1]. Cigarette smoking is the most significant risk factor for developing lung cancer and contributes to 80–90% of these deaths [2, 3]. 

Over the past four to five decades, significant progress has been made to elucidate the carcinogenic mechanisms of tobacco smoking. Using animal models, it has been shown that among over 60 established carcinogens in cigarette smoke, 20 can cause lung tumors [4]. It has been proposed that these carcinogens, when metabolized, form DNA-adducts which may directly cause genetic alterations if not repaired. When these genetic alterations affect tumor suppressor genes or tumor oncogenes, they can promote cell proliferation and malignant transformation [5]. Studies in lung cancer patients clearly suggest that cigarette smoking can lead to acquisition of genetic mutations in p53 and ras oncogene [6, 7]. In addition, cigarette smoke is proposed to cause immunosuppression, which provides an environment for tumor progression [8, 9]. 

Recently, DNA hypermethylation has been recognized as an alternative, epigenetic mechanism for gene silencing in lung cancer, in addition to genetic mutation. Several environmental exposures are thought to cause aberrant DNA methylation, including dietary factors, chemotherapeutic agents, and heavy metals [10]. Tobacco smoke exposure has been associated with increased expression of DNA methyltransferases [11–14]. Consistent with this observation, lung cancers arising in heavy smokers show increased hypermethylation of various genes, especially CDKN2A (p16) and RASSF1, compared with lighter smokers or nonsmokers [15–27]. 

However, these results do not reveal whether DNA hypermethylation occurs early or late in carcinogenesis. Early changes in carcinogenesis (especially those related to smoking) are hypothesized to occur somewhat diffusely in the lung and may therefore be detectable in noncancerous lung tissue, as well as in any cancers which arise [28–30]. For example, frequent hypermethylation of CDKN2A, RASSF1, CDH13, and other genes has been observed in sputum samples from cancer-free smokers, suggesting that they may be hypermethylated early [31–34]. In contrast, late changes in carcinogenesis are thought to arise mainly in overtly malignant tissues.

We recently analyzed matched cancerous and noncancerous lung tissues from patients with nonsmall cell lung cancer (NSCLC). We observed that in the 27 genes tested, most DNA methylation changes were tumor-specific and therefore might be considered late changes in carcinogenesis [35]. However, in these NSCLC patients, a small number of genes, including CCND2, APC, CDH1, and RARB (Table 1), were also hypermethylated in a portion of noncancerous lung tissues, suggesting that one or more of these genes might become hypermethylated as an early precancerous change. We hypothesized that early changes in DNA methylation, if present, might be associated with exposure to cigarette smoke. Furthermore, because smoking-related lung tumors and emphysema are known to disproportionately affect the upper lobes of the lungs [36, 37], we hypothesized that methylation changes related to smoking would similarly be more frequent in the upper lobes, compared with the lower lobes.

## 2. Materials and Methods

### 2.1. Subject Enrollment

All procedures were conducted in accordance with institutional review board and human subjects committee approval. Subjects were retrospectively enrolled who had undergone lung surgeries (lung volume reduction, lung transplant, wedge biopsy, or lobectomy) for nonmalignant diseases including emphysema, chronic bronchitis, bronchiectasis, granulomatous disease, various infectious diseases, and cystic or pulmonary fibrosis, at the University of Washington Medical Center (UWMC) between July 1st 1995 and July 1st 2005. All specimens were reviewed by an expert pathologist (CDJ) to confirm that they represented noncancerous lung tissue. All clinical data were gathered from subjects' UWMC medical records, including smoking history and primary pulmonary diagnosis. Subjects were excluded for the following reasons: previous diagnosis of lung cancer, insufficient lung tissue for methylation analysis, or unknown pack years of smoking. In total, 372 nonmalignant lung tissue samples from 159 subjects were identified for DNA methylation analysis.

### 2.2. DNA Isolation from Paraffin Blocks

From each block, six 20-*μ*m sections were cut and deparaffined by xylene extraction. Proteinase K was used to digest the resulting tissue pellets overnight, at 48°C. Genomic DNA was then isolated by phenol/chloroform extraction and ethanol precipitation. Finally, DNA was purified using a QIAamp DNA minicolumn (Qiagen) according to the manufacturer's instructions.

### 2.3. Sodium Bisulfite Conversion

As previously described in detail [42], *in vitro* fully methylated DNA (methylated DNA control) and human sperm DNA (unmethylated DNA control) were converted with clinical samples. Briefly, *∼*1 *μ*g DNA was modified by 5 mol/L sodium bisulfite, desulfonated with NaOH, and then purified and resuspended in 80 *μ*L elution buffer (EB; 10 mmol/L Tris-HCl, pH 8.0).

### 2.4. DNA Methylation (MethyLight) Assay

All primers and probes for MethyLight assays were designed specifically for bisulfite-converted fully methylated DNA. Their sequences have been reported previously [35]. Amplification of bisulfite converted beta-actin (ACTB) DNA was used to normalize for the quantity of input DNA. Samples negative for ACTB were excluded from methylation analysis. Of 372 identified samples, 56 (15%) were excluded because they were negative for ACTB. The percentage of samples excluded after bisulfite conversion was similar in smokers (15%) and nonsmokers (16%). A plasmid containing bisulfite converted ACTB gene of known concentration was diluted and used as a standard curve for quantification. The assay for a given set of samples was only considered valid if the converted unmethylated human sperm DNA was not amplified, whereas the converted fully methylated DNA was amplified. For each locus, the percentage methylated reference (PMR) was calculated by dividing the gene/reference ratio of a sample by the gene/reference ratio of fully methylated DNA control [43]. Genes were considered to be positive for any hypermethylation at PMR >0%.

### 2.5. Statistical Methods

For comparisons between groups, to provide independent observations, we randomly selected one tissue block per subject to represent each subject's hypermethylation profile. To evaluate potential differences in gene methylation by site of the lung, paired upper, and lower lobe tissue samples from within subjects were compared using McNemar's Test. To assess the univariate and multivariate relationships between gene methylation and independent variables (smoking, age, gender, lobe of lung, pack years, and years since quitting), we included all available tissue samples from each subject and employed generalized estimating equations (GEE). This method enables the analysis of data with repeated measurements (multiple tissue samples per subject from different lobes) and accounts for within-subject correlations. In selecting a model, a logit link was used and we assumed an exchangeable working correlation structure to account for intrasubject correlation. Parameter estimates were exponentiated to provide odds ratios (OR) and 95% confidence intervals (CI). A 2-sided 0.05 test level determined statistical significance for all analyses. All analyses were conducted using SAS version 9.1 (SAS Institute Inc., Cary, NC).

## 3. Results

### 3.1. Study Population and Tissue Samples

We retrospectively enrolled 151 subjects who contributed a total of 316 available pathology blocks (Table 2). At the time of their surgery, 121 subjects were current or former smokers (ever-smokers), while 30 reported no smoking history (never-smokers). Among the never-smokers, none had any history of cancer, either prior to or after the surgery that yielded the tissue used in this study. Of the ever-smokers, 10 had a history of prior cancer other than lung (1 breast, 2 cervical, 2 colon, 2 prostate, 1 testicular, and 2 uterine) and all were cancer free at surgery. Four of the ever-smokers developed a cancer subsequent to the surgery that yielded the cancer-free lung tissue (1 bladder, 1 colon, and 2 NSCLCs, one at 2 years after, one at 5 years after). The clinical data show that never-smokers and ever-smokers who had undergone lung surgery comprised two distinct populations. Ever-smokers were significantly older than never-smokers (61 years versus 44 years). Further, of ever-smokers who contributed specimens from lung surgery, 71% had a diagnosis of emphysema, compared to only 10% of never-smokers. 

We analyzed a total of 316 available pathology blocks from these 151 subjects, including 177 upper lobe samples, 105 lower lobe samples, 30 middle lobe or lingula samples, and 4 whose lobe of origin was unclear. Multiple blocks were available for 98 (81%) of ever-smokers and 13 (43%) of never-smokers; from the 121 ever-smokers, 269 samples were tested, while from the 30 never-smokers, 47 samples were tested. Sample sites varied substantially in ever-smokers and never-smokers as 50% of ever-smokers, compared to 17% of never-smokers, contributed only samples from the upper lobes. This difference arose because many ever-smokers in our sample underwent lung volume reduction surgery for emphysema, which predominantly affects the upper lung zones when induced by smoking.

### 3.2. Gene Hypermethylation and Smoking Status

**Figure 1:**
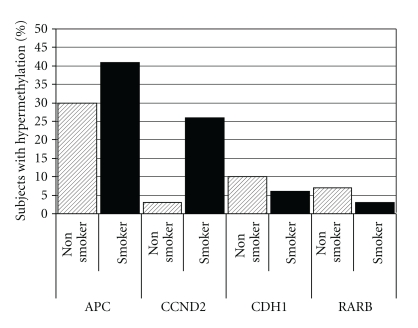
Hypermethylation of four genes in noncancerous lung tissues. Percent of subjects with hypermethylation of four genes (APC, CCND2, CDH1, and RARB), stratified by smoking status. Samples were considered to be positive for any hypermethylation at PMR >0%. To provide population statistics, one lung tissue sample per subject was randomly selected. The 15 other genes tested were hypermethylated in <2% of all subjects.

Considering one random tissue block per subject, only APC (39%), CCND2 (21%), CDH1 (7%), and RARB (4%) were hypermethylated in more than 2% of subjects (Figure 1). All 15 remaining genes (BVES, CDH13, CDKN2A (p16), CDKN2B, DAPK1, IGFBP3, IGSF4, KCNH5, KCNH8, MGMT, OPCML, PCSK6, RASSF1, RUNX, and TMS1) were hypermethylated in less than 2% of subjects. CCND2 was hypermethylated significantly more frequently in ever-smokers compared to never-smokers (26% versus 3%, *P* < .001). APC was hypermethylated somewhat more frequently in ever-smokers (41% versus 30%), but this did not achieve statistical significance (*P* = .3).

### 3.3. Correlation of APC and CCND2 Gene Hypermethlation

APC and CCND2 were often hypermethylated in the same samples; 179 (57%) samples were negative for both genes, 16 (5%) were positive for hypermethylation of CCND2, but not APC, 68 (22%) were positive for hypermethylation of APC but not CCND2, and 53 (17%) samples were positive for both genes. CCND2 hypermethylation was significantly correlated with APC hypermethylation in all subjects (OR = 7.3, 95% CI = 3.9–13.8) and in smokers only (OR = 7.4, 95% CI = 3.9–14.0). In nonsmokers, 31 (66%) samples were negative for both genes, 14 (30%) were positive for APC only, and 2 (4%) samples were positive for both APC and CCND2.

### 3.4. Gene Hypermethylation and Clinical and Demographic Factors

In univariate GEE analyses of all specimens (Table 3), CCND2 hypermethylation was significantly associated with a positive smoking history, increasing age, and sample origin from the upper versus lower lobe of the lung. APC hypermethylation was significantly less frequent among females and moderately more frequent in upper lobes compared to lower lobes but was not significantly associated with a positive smoking history. In a multivariate model simultaneously assessing smoking history, age, gender, and location of the sample (upper versus lower lobe) in all subjects, hypermethylation of CCND2 remained significantly associated with increased age (OR = 1.7, 95% CI = 1.2–2.4 for each 10 years of age) and upper lobe location (OR = 2.0, 95% CI = 1.0–3.8). CCND2 hypermethylation was somewhat associated with a positive smoking history (OR = 2.8, 95% CI = 0.6–12.1) but this did not achieve statistical significance.

### 3.5. Gene Hypermethylation and Duration of Smoke Exposure

Within the subset of 269 samples from 121 ever-smokers (Table 4), APC hypermethylation was not related to pack-years of cigarette smoking or years since quitting smoking. In univariate GEE analysis, CCND2 hypermethylation was significantly associated with greater pack years but was not related to years since quitting. However, in a multivariate GEE analysis simultaneously assessing pack years, years since quitting, age, gender, and location of the sample (upper versus lower lobe), CCND2 hypermethylation was no longer associated with pack years (OR = 1.0, 95% CI = 0.9–1.2 per 10 pack years).

### 3.6. Gene Hypermethylation in Upper- and Lower-Lobe Samples

Smoking-related lung tumors and emphysema are known to disproportionately affect the upper lobes of the lungs. Thus, if hypermethylation of a gene is associated with smoking, we might expect to find more hypermethylation in upper lobe samples compared to lower lobe samples, among ever-smokers. 

Examining all 269 samples from ever-smokers (Table 4), in univariate GEE analysis, both APC (OR = 2.0, 95% CI = 1.1–3.5) and CCND2 (OR = 1.9, 95% CI = 1.0–3.5) hypermethylation were more common in upper compared to lower lobes. In multivariate analysis including pack-years, years since quitting, age, gender, and upper versus lower lobe, APC hypermethylation remained significantly associated with upper-lobe sample location (OR = 2.1, 95% CI = 1.1–4.0), while CCND2's positive association with upper lobes was reduced to slightly below the level of statistical significance (OR = 1.7, 95% CI = 0.9–3.4).

Among the 121 ever-smokers in our cohort, 30 had both upper and lower lobe samples available, and were included in within-subjects, pairwise comparisons. For APC, 12 of 30 pairs had discordant hypermethylation status (1 positive and 1 negative), of which 8 of 12 displayed APC hypermethylation in an upper lobe but not a lower lobe sample (*P* = .25). For CCND2, only 7 of 30 pairs had discordant hypermethylation status, of which only 3 of 7 were hypermethylated in the upper but not the lower lobe (*P* = .7). Thus, too few subjects had discordant hypermethylation in upper and lower lobe samples to yield statistically meaningful results in within-subjects comparisons.

## 4. Discussion

DNA hypermethylation is an important event in lung carcinogenesis. However, it is currently unknown whether changes in DNA methylation are early events, occurring in previously normal lung tissue or whether they are late changes that occur only in overt tumor cells [30]. To attempt to answer these questions, we tested DNA hypermethylation in lung tissues from subjects without cancer—both smokers and nonsmokers—using a panel of 19 genes which we had previously found to be hypermethylated in some nonsmall cell lung cancers [35, 44]. This unique study design allowed us, for the first time, to characterize the DNA hypermethylation profile of nonsmokers' lung tissues and to compare this profile to that of smoke-exposed lung.

Importantly, we observed that CCND2, which is known to be frequently hypermethylated in lung cancer tissue [35, 44–47], was hypermethylated more frequently in ever-smokers (26%) than in never-smokers (3%). Also, as predicted, in ever-smokers, CCND2 was hypermethylated more frequently in samples from the upper lobes, which are known to suffer far more negative effects from cigarette smoke, such as lung cancer and emphysema [36, 37]. These findings support the conclusion that CCND2 reflects an early, precancerous change in the lung, caused by cigarette smoke.

CCND2 encodes cyclin D2, a protein involved in cell cycle progression that is thought to act as a regulator of cyclin dependent kinase 4 and cyclin dependent kinase 6 in the transition from G1 to S-phase [39]. CCND2 hypermethylation appears to be common in many cancers. In breast cancer, where it has been studied most extensively, CCND2 hypermethylation is detected frequently, though it appears to be rarely detected in normal breast tissue [48–54]. Interestingly, while CCND2 hypermethylation (and therefore low CCND2 protein expression) has been associated with poor prognosis in epithelial ovarian cell cancer [55] and recurrence of hepatocellular carcinoma [56], *increased* CCND2 expression has been associated with poor prognosis in diffuse large B-cell lymphoma [57].

In the lung, CCND2 hypermethylation has been found in 40–56% of NSCLCs [35, 44, 45, 47]. In noncancerous lung tissue, whereas Virmani et al. found CCND2 hypermethylation in 0 of 18 samples [45], our previous investigation found CCND2 hypermethylation in 24% of noncancerous lung tissues from patients with NSCLC [35]. This closely matches the rate observed in the present study, in cancer-free ever-smokers (26%). Possibly, our group observed a higher rate of CCND2 hypermethylation in both cancerous and cancer-free lung tissues because we used MethyLight assays instead of methylation-specific PCR (MSP), which was used by Virmani et al. Thus, we may have detected low levels of hypermethylated genes in cancer-free tissues which were not detected by MSP. Discrepancies may also be due to the somewhat different primers and probes used in analyses, which indicate different sequence regions investigated. In addition, Kubo et al. did not observe any CCND2 hypermethylation in 30 matched noncancerous lung tissues but it should be noted that in this study, 70% of subjects were nonsmokers who would not be expected to have significant rates of CCND2 hypermethylation [46]. 

Combined, these results reveal a progression in the rate of CCND2 hypermethylation in the lung, corresponding with the risk for developing lung cancer. While CCND2 hypermethylation was very infrequent (3%) in our current study's low-risk group of 30 never-smokers, it was more frequent in a high-risk group of ever-smokers (24–26% in our current and previous studies), and most frequent in overt NSCLC tissue (40–56%). This risk-stratified progression in lung tissues suggests that CCND2 hypermethylation may truly reflect an early precancerous change in the lung, en route to overt cancer, which may be due to the effects of smoking. 

Still, our findings regarding CCND2 should be regarded as preliminary at this time, for several reasons. In multivariate analysis, the effect of smoking status on CCND2 hypermethylation was reduced to trend-level significance after taking into account the effects of sample location (upper versus lower lobe) and subject age. This likely occurred because in our sample, the majority of smokers underwent lung surgery for emphysema and represented a significantly older group, more likely to contribute samples from upper lobes (where emphysema is most prominent). In contrast, nonsmokers were younger and underwent lung resection for a variety of diseases. With such significant correlation of these factors, multivariate analysis may not have reliably separated each factor's relative contribution to gene hypermethylation. Thus, observed differences in the rate of CCND2 hypermethylation could be attributable to any of these factors or others that differed between ever and never-smokers. Emphysema, for example, made CCND2 hypermethylation more likely although significant rates of CCND2 hypermethylation were also found in smokers with other diagnoses. While CCND2 hypermethylation could be part of the unique pathophysiology of emphysema, it more likely arose because emphysema reflects severe smoking-induced lung damage. The effect of age on CCND2 hypermethylation has not been studied previously in noncancerous lung, although several genes have been reported to undergo increased rates of hypermethylation with age, in various body tissues, including CDH1 and DAPK1 in the lung [58]. In noncancerous breast epithelium [59] and in peripheral blood samples from cancer-free subjects [60], advanced age was not observed to correlate with CCND2 hypermethylation. Thus, the relationship between age and CCND2 hypermethylation remains unknown at this time. In weighing the relative contributions of age, sample location, and emphysema status on CCND2 hypermethylation, it is worth noting that smoking history was by far the strongest single predictor of CCND2 hypermethylation in univariate analysis (OR = 6.9, 95% CI = 1.6–29.8). One limitation of the present study was that despite our overall large number of 151 subjects, only 30 were never-smokers. This occurred because never-smokers far less frequently undergo lung resections which produce tissue. This may have been part of the reason why in multivariate analysis, we observed only trend-level significance for smoking's effect on CCND2 hypermethylation. We were able to improve our statistical power somewhat by using generalized estimating equations (GEE) for our univariate and multivariate analyses, allowing us to enter multiple tissue blocks per subject when available (multiple observations), without biasing the results. However, future studies should seek to verify the low rate of CCND2 hypermethylation we observed in never-smokers. An additional limitation of our study design was that all subjects had an underlying non-cancer pulmonary diagnosis that necessitated lung surgery. Thus, while observed gene hypermethylation was unrelated to cancer, it cannot definitely be said to represent healthy lung. Finally, due to our study design, we only provide indirect evidence of interaction between smoking and CCND2 hypermethylation. Future studies utilizing animal models may be useful to elucidate the potential causal relationship between smoking and CCND2 hypermethylation. 

In our current and previous studies, CDKN2A (p16) was hypermethylated in 26% of cancer tissues [44] but was rarely hypermethylated in noncancerous lung tissues, regardless of smoking status [35]. However, CDKN2A hypermethylation has previously been characterized as an early event in lung carcinogenesis [28–30], and hypermethylation of CDKN2A has been commonly detected in sputum samples from heavy smokers without lung cancer [32, 61]. Overall, a very wide range of hypermethylation rates for CDKN2A has been reported in the literature, for noncancerous lung tissues. Along with other researchers who observed low rates of CDKN2A hypermethylation in noncancerous lung tissues, our results suggest that CDKN2A hypermethylation may actually represent a later change in carcinogenesis [62–66]. However, the surprisingly large discrepancies between studies may be related to differences in assay methodology (including PCR primers and specific CpG islands) or patient populations.

## 5. Conclusions

CCND2 hypermethylation likely represents an early, smoking-induced, precancerous change in the lung; it is very infrequent in the lung tissue of never-smokers, more frequent among smokers, and most frequent in overt NSCLC tissue. This conclusion should be verified in future investigations. In addition, this study supports the conclusions of our previous investigation, that although they are hypermethylated in many NSCLC tumor tissues, RASSF1, DAPK1, BVES, CDH13, MGMT, KCNH5, and to some extent CDH1 and RARB, are rarely hypermethylated in the cancer-free lung, even after significant tobacco exposure [35]. These genes may therefore yield clues to understanding the later stages of carcinogenesis. In addition, if hypermethylation of CCND2 or other genes represents an early precancerous change, it is possible that drugs aimed at reversing DNA methylation could be used to prevent smoking-related carcinogenesis.

##  Conflict of Interests 

None Declared.

## Figures and Tables

**Table 1:** Genes hypermethylated in >2% of noncancerous lung tissues.

HUGO acronym	Gene name	Function
APC	Adenomatous polyposis coli	Cell cycle: inhibits WNT signaling pathway, involved in spindle assembly and chromosome segregation, cell adhesion, and cell migration [38].
CCND2	Cyclin D2	Cell cycle: regulates entry into S-phase with CDK4 and CDK6 [39]
CDH1	Cadherin 1; e-cadherin (epithelial)	Cell adhesion, epithelial-mesenchymal transition [40]
RARB	Retinoic acid receptor, beta	Regulation of cell proliferation and differentiation [41]

**Table 2:** Clinical data of 151 cancer-free subjects with lung tissue available for MethyLight assay.

	Never-smokers (*n* = 30)	Ever-smokers (*n* = 121)
*Age at surgery (mean years ± sd)*	43.7 ± 11.6	61.0 ± 9.9
20–39	11 (37%)	2 (2%)
40–49	8 (27%)	14 (12%)
50–59	9 (30%)	33 (27%)
60–69	2 (7%)	43 (36%)
70–79	0 (0%)	29 (24%)

*Female gender*	18 (60%)	58 (48%)

*Smoking pack years*		
1–39	N/A	50 (41%)
≥40	N/A	71 (59%)

*Years since quitting* ^ a^		
0 (Current)	N/A	15 (13%)
1–4	N/A	31 (26%)
5–9	N/A	27 (23%)
10–19	N/A	30 (25%)
≥20	N/A	17 (14%)

*Surgery*		
Lung volume reduction	0 (0%)	57 (47%)
Lung transplant	9 (30%)	31 (26%)
Wedge Biopsy	18 (60%)	24 (20%)
Lobectomy	2 (7%)	5 (4%)
Bullectomy	0 (0%)	4 (3%)
Segmentectomy	1 (3%)	0 (0%)

*Number of samples evaluated*		
One sample	17 (57%)	23 (19%)
Multiple samples	13 (43%)	98 (81%)

*Sample locations* ^ b^		
Upper lobe only	5 (17%)	60 (50%)
Middle lobe or lingula only	6 (21%)	1 (1%)
Lower lobe only	10 (34%)	21 (18%)
Multiple lobes	8 (28%)	37 (31%)

*Etiology*		
Emphysema^c^	3 (10%)	86 (71%)
Inflammatory conditions^d^	13 (43%)	21 (17%)
Infectious diseases	4 (13%)	7 (6%)
Cystic fibrosis	5 (17%)	0 (0%)
Pulmonary hypertension	1 (3%)	2 (2%)
Sarcoidosis	1 (3%)	1 (1%)
Lymphoid hyperplasia	1 (3%)	1 (1%)
Infarct	1 (3%)	1 (1%)
Hemangioma	0 (0%)	1 (1%)
Trapped lung	1 (3%)	0 (0%)
No histologic abnormalities	0 (0%)	1 (1%)

^a^Quit years not available for 1 subject.

^b^Sample location unknown for 4 samples from 3 subjects.

^c^See results section for details.

^d^Inflammatory conditions included chronic bronchitis, bronchiectasis, pulmonary fibrosis, and granulomatous disease.

**Table 3:** All subjects—odds ratios for promoter hypermethylation (95% CI).

	Ever-smokers versus never-smokers	Age per 10 years	Female versus male	Upper versus lower lobe
Univariate^a^				
APC	1.3 (0.6–2.9)	1.1 (0.9–1.4)	0.6 (0.5–0.8)	1.6 (0.9–2.7)
CCND2	6.9 (1.6–29.8)	1.9 (1.4–2.7)	0.8 (0.6–1.1)	2.3 (1.2–4.4)
Multivariate^a^				
APC	1.0 (0.4–2.6)	1.0 (0.8–1.3)	0.6 (0.4–0.8)	1.6 (1.0–2.8)
CCND2	2.8 (0.6–12.1)	1.7 (1.2–2.4)	0.8 (0.6–1.2)	2.0 (1.0–3.8)

^a^Associations between clinical parameters and gene hypermethylation, assessed at PMR > 0%, in all 316 lung specimens from 151 subjects.

**Table 4:** Ever-smokers only—odds ratios for promoter hypermethylation (95% CI).

	Pack years per 10 years	Quit years per 10 years	Age per 10 years	Female versus male	Upper versus lower lobe
Univariate^a^					
APC	1.0 (0.9–1.1)	0.9 (0.6–1.2)	1.1 (0.8–1.5)	0.6 (0.5–0.8)	2.0 (1.1–3.5)
CCND2	1.1 (1.0–1.3)	0.8 (0.6–1.1)	1.8 (1.3–2.6)	0.8 (0.6–1.1)	1.9 (1.0–3.5)
Multivariate^a^					
APC	0.9 (0.8–1.1)	0.8 (0.6–1.2)	1.2 (0.8–1.7)	0.6 (0.4–0.8)	2.1 (1.1–4.0)
CCND2	1.0 (0.9–1.2)	0.8 (0.5–1.2)	1.8 (1.2–2.9)	0.9 (0.6–1.2)	1.7 (0.9–3.4)

^a^Associations between clinical parameters and gene hypermethylation in 269 lung specimens from 121 subjects with a current or past history of smoking.
